# Quality of life changes: a prospective evaluation after surgery ablation atrial fibrillation

**DOI:** 10.1186/1749-8090-10-S1-A254

**Published:** 2015-12-16

**Authors:** Alexander Chernyavskiy, Yulia Kareva, Inessa Pak, Sardor Rakhmonov, Evgeny Pokushalov, Alexander Romanov, Sergey Alsov

**Affiliations:** 1 Department of Surgery, Aorta, Coronary and Peripheral Arteries, State Research Institute of Circulation Pathology, Novosibirsk, 630055, Russia; 2 Arrhythmia Department and Electrophysiology Laboratory, State Research Institute of Circulation Pathology, Novosibirsk, 630055, Russia

## Background/Introduction

Atrial fibrillation (AF) is a chronic condition that increases the risk of patient mortality and morbidity and often requires life-long treatment, including long-term oral anticoagulation. Therefore, quality of life (QoL) is an important treatment outcome when measuring patients' physical, emotional, and social functioning, as well as their perceived health.

## Aims/Objectives

Compare the quality of life (QoL) of patients with persistent atrial fibrillation (pAF) and coronary artery disease (CAD) after modified mini-maze (MM) procedure or pulmonary vein isolation (PVI) using irrigated radiofrequency ablation (irrRA) compared with patients in the control group (CABG alone).

## Method

In this prospective randomized study, we included 95 patients with pAF and CAD who underwent CABG combined with intraoperative irrRA. Patients were randomly assigned to 3 groups: CABG and PVI (CABG+PVI, n = 31), CABG and MM procedure (CABG+MM, n = 30), and isolated CABG (CABG alone, n = 34). All patients received implantable loop recorders (ILRs). Patient QoL was assessed using the Short Form 36 (SF-36) preoperatively, and 1 and 2 years postoperatively. The study primary end point- freedom from AF 1 year postoperatively, measured by ILRs; secondary end point - long-term outcomes, QoL, mortality, cardiovascular events.

## Results

No reoperations or hospital mortalities were recorded. Mean follow-up was 14.4 ± 9.7 months. The freedom from AF were 80%, 86.2%, and 44.1% in the CABG+PVI, CABG+MM, and in the CABG alone groups, respectively. The QoL significantly improved in CABG+PVI and CABG+MM groups compared with CABG alone group in most domains.

## Discussion/Conclusion

Effective elimination of AF during CABG surgery improves QoL in all physical health domains of the SF-36 and the role-emotional functioning domain. Thus, patients with concomitant AF and CAD may benefit from intraoperative irrRA to prevent relapse of the arrhythmia.

